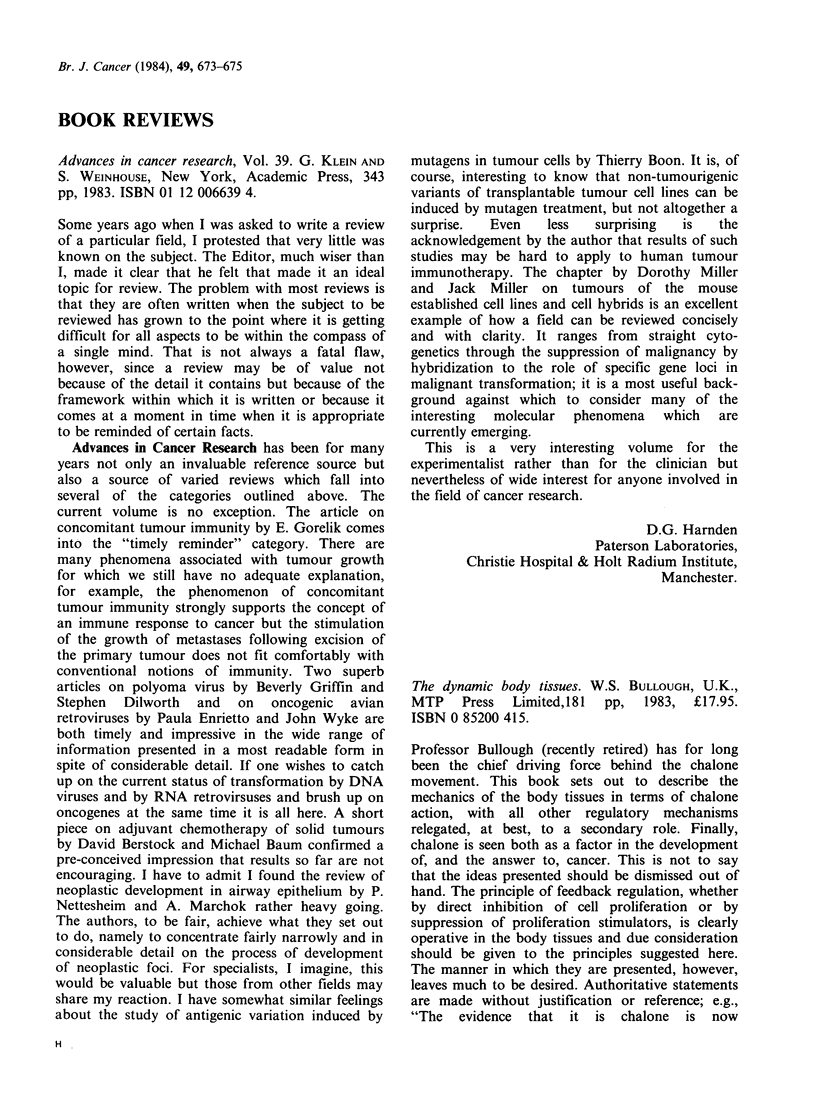# Advances in cancer research

**Published:** 1984-05

**Authors:** D.G. Harnden


*Br. J. Cancer* (1984), **49**, 673–675

### BOOK REVIEWS

*Advances in cancer research*, Vol. 39. G. KLEIN AND S. WEINHOUSE, New York, Academic Press, 343 pp, 1983. ISBN 01 12 006639 4.

Some years ago when I was asked to write a review of a particular field, I protested that very little was known on the subject. The Editor, much wiser than I, made it clear that he felt that made it an ideal topic for review. The problem with most reviews is that they are often written when the subject to be reviewed has grown to the point where it is getting difficult for all aspects to be within the compass of a single mind. That is not always a fatal flaw, however, since a review may be of value not because of the detail it contains but because of the framework within which it is written or because it comes at a moment in time when it is appropriate to be reminded of certain facts.

**Advances in Cancer Research** has been for many years not only an invaluable reference source but also a source of varied reviews which fall into several of the categories outlined above. The current volume is no exception. The article on concomitant tumour immunity by E. Gorelik comes into the "timely reminder" category. There are many phenomena associated with tumour growth for which we still have no adequate explanation, for example, the phenomenon of concomitant tumour immunity strongly supports the concept of an immune response to cancer but the stimulation of the growth of metastases following excision of the primary tumour does not fit comfortably with conventional notions of immunity. Two superb articles on polyoma virus by Beverly Griffin and Stephen Dilworth and on oncogenic avian retroviruses by Paula Enrietto and John Wyke are both timely and impressive in the wide range of information presented in a most readable form in spite of considerable detail. If one wishes to catch up on the current status of transformation by DNA viruses and by RNA retrovirsuses and brush up on oncogenes at the same time it is all here. A short piece on adjuvant chemotherapy of solid tumours by David Berstock and Michael Baum confirmed a pre-conceived impression that results so far are not encouraging. I have to admit I found the review of neoplastic development in airway epithelium by P. Nettesheim and A. Marchok rather heavy going. The authors, to be fair, achieve what they set out to do, namely to concentrate fairly narrowly and in considerable detail on the process of development of neoplastic foci. For specialists, I imagine, this would be valuable but those from other fields may share my reaction. I have somewhat similar feelings about the study of antigenic variation induced by mutagens in tumour cells by Thierry Boon. It is, of course, interesting to know that non-tumourigenic variants of transplantable tumour cell lines can be induced by mutagen treatment, but not altogether a surprise. Even less surprising is the acknowledgement by the author that results of such studies may be hard to apply to human tumour immunotherapy. The chapter by Dorothy Miller and Jack Miller on tumours of the mouse established cell lines and cell hybrids is an excellent example of how a field can be reviewed concisely and with clarity. It ranges from straight cytogenetics through the suppression of malignancy by hybridization to the role of specific gene loci in malignant transformation; it is a most useful background against which to consider many of the interesting molecular phenomena which are currently emerging.

This is a very interesting volume for the experimentalist rather than for the clinician but nevertheless of wide interest for anyone involved in the field of cancer research.

D.G. Harnden
Paterson Laboratories,
Christie Hospital & Holt Radium Institute,
Manchester.